# Methodology for computing the burden of disease of adverse events following immunization

**DOI:** 10.1002/pds.4419

**Published:** 2018-03-24

**Authors:** Scott A. McDonald, Danielle Nijsten, Kaatje Bollaerts, Jorgen Bauwens, Nicolas Praet, Marianne van der Sande, Vincent Bauchau, Tom de Smedt, Miriam Sturkenboom, Susan Hahné

**Affiliations:** ^1^ National Institute for Public Health and the Environment (RIVM) Bilthoven The Netherlands; ^2^ P95 Pharmacovigilance and Epidemiology Services Leuven Belgium; ^3^ University of Basel Children's Hospital Basel Switzerland; ^4^ Brighton Collaboration Foundation Basel Switzerland; ^5^ GlaxoSmithKline Vaccines Rixenart Belgium; ^6^ Department Public Health Institute of Tropical Medicine Antwerp Belgium; ^7^ VACCINE.GRID Foundation Basel Switzerland

**Keywords:** adverse events, disability‐adjusted life‐years, disease burden, methodology, pharmacoepidemiology, vaccination

## Abstract

**Purpose:**

Composite disease burden measures such as disability‐adjusted life‐years (DALY) have been widely used to quantify the population‐level health impact of disease or injury, but application has been limited for the estimation of the burden of adverse events following immunization. Our objective was to assess the feasibility of adapting the DALY approach for estimating adverse event burden.

**Methods:**

We developed a practical methodological framework, explicitly describing all steps involved: acquisition of relative or absolute risks and background event incidence rates, selection of disability weights and durations, and computation of the years lived with disability (YLD) measure, with appropriate estimation of uncertainty. We present a worked example, in which YLD is computed for 3 recognized adverse reactions following 3 childhood vaccination types, based on background incidence rates and relative/absolute risks retrieved from the literature.

**Results:**

YLD provided extra insight into the health impact of an adverse event over presentation of incidence rates only, as severity and duration are additionally incorporated. As well as providing guidance for the deployment of DALY methodology in the context of adverse events associated with vaccination, we also identified where data limitations potentially occur.

**Conclusions:**

Burden of disease methodology can be applied to estimate the health burden of adverse events following vaccination in a systematic way. As with all burden of disease studies, interpretation of the estimates must consider the quality and accuracy of the data sources contributing to the DALY computation.

**KEY POINTS**

- Burden of disease measures such as the disability‐adjusted life‐year (DALY) are frequently employed to quantify population‐level disease impact, and effects of interventions.
- DALY methodology is also applicable to adverse events following vaccination, usefully combining event incidence, severity, and duration in a single composite measure.
- By means of a worked example, we describe the computations and data sources required to compute DALYs for adverse events and identify the most probable information gaps.

## 1 INTRODUCTION

Vaccination is indisputably recognized as one of the foremost public health interventions developed within the last century. Despite the drastic improvements in population health attributed to vaccination, there has been public concern regarding possible negative consequences from being vaccinated, namely adverse events.^1^ The WHO defines adverse events following immunization (AEFI) as “any untoward medical occurrence which follows immunization and which does not necessarily have a causal relationship with the usage of the vaccine”.^2^ All AEFI represent reductions in one's current health status. In the present paper, we focus on *adverse reactions*; these are AEFI which have an identified, well‐recognized increased risk of occurrence following vaccination.

For the measurement of health burden, composite burden of disease (BoD) measures—such as the disability‐adjusted life‐years (DALY) measure—have been developed.^3^ To date, there has been limited assessment of the population‐level health burden of AEFI using BoD measures.^4^, ^5^ Although selected safety aspects of vaccination have a long history of investigation, studies are often limited to rates or risk estimates. A comprehensive estimate of the population‐level health burden of events associated with vaccination and the extent of the AEFI burden relative to the disease being prevented through vaccination would be useful contributions to current knowledge. In addition, quantitative estimates of AEFI burden fit well within the scope of benefit‐risk methodology^6^ for assessment of new or existing vaccines, if the (projected) averted disease burden due to vaccination can also be quantified.^7^, ^8^

A quantitative measure of health burden should ideally take into account the frequency of occurrence, severity and duration of illness, the risk of eventual complications, and the risk of mortality, to allow meaningful comparison between heterogeneous conditions and their effects on the full spectrum of health. The utility of summary measures of population health goes beyond that of simple epidemiological indicators such as incidence or mortality rates, as they integrate mortality with morbidity in a single indicator—taking into account both severity and duration of illness/disability—and therefore are suitable for making comparisons between events, vaccine types, age groups, and national or regional populations. The DALY is a commonly used summary measure of population health burden^3^, ^9^, ^10^ and is typically applied to compare the relative impact of diseases on a population. Composite burden measures such as the DALY additionally offer a common currency for representing both beneficial (eg, DALYs averted due to a preventive measure such as vaccination) and detrimental (eg, DALYs lost due to AEFI) impacts on health, which is desirable for benefit‐risk assessment.

Our principal objective was to assess the feasibility of adapting current disease burden methodology for computing the population‐level disease burden of vaccination‐attributable adverse events. This work was carried out as part of the “Accelerated development of vaccine benefit‐risk collaboration in Europe” (ADVANCE) project, launched in 2013, funded by the Innovative Medicines Initiative (http://www.advance-vaccines.eu). The aim of ADVANCE is to help health professionals, regulatory agencies, public health institutions, vaccine manufacturers, and the general public make well‐informed and timely decisions on benefits and risks of marketed vaccines by establishing a framework and toolbox to enable rapid delivery of reliable data on vaccine benefits and risks.

## 2 METHODS

We piloted our methodological approach using a selected set of events well recognized as adverse reactions following immunization. Selection was based on the frequency of occurrence and potential severity of the event. Certain events might occur relatively frequently, but although mild can still be responsible for causing disability (ie, vaccination recipient experiences less than full health), or they might occur extremely rarely, but with serious consequences. We estimated the disease burden, in DALYS, for example events falling within both broad categories of AEFI: (1) infrequent, but potentially serious events (idiopathic thrombocytopenic purpura [ITP], anaphylaxis); and (2) relatively frequent, but less serious events (febrile convulsions) (Table 1). The burden computation was then restricted to only those vaccine‐event pairs with a likelihood of a strong association (based on the Global Research in Pediatrics [GRiP] evaluation of strength of evidence; ie, vaccine‐event pair either identified as a “positive control”,^19^ or reported by the Institute of Medicines^20^). Below, we describe the methodology and apply it to a worked example.

**Table 1:** Selected adverse events and sources for event incidence rates

Adverse Event	Category [Frequency/ Severity]	Age Group	Background Incidence Rate (95% CI)	Period and Setting	Reference
Idiopathic thrombo‐cytopenic purpura	Infrequent/ high	<2 yrs 2–5 yrs	6.8/100 000 (4.9–9.2) 7.2/100 000 (5.9–8.8)	1990–2005, UK	Yong et al, 2010^11^
Anaphylaxis	Infrequent/ high		*Alternative approach used ‐ see* Table 2		
Febrile convulsions	Frequent/low	2–12 mos	556/100 000 (537–575)	1999–2011, UK	Sammon et al, 2015^18^
		13–24 mos	1377/100 000 (1348–1407)		
		25–60 mos	432/100 000 (413–433)		
		61–120 mos	58/100 000 (54–61)		
		121–180 mos	23/100 000 (18–28)		

### 2.1 Burden of disease methods and required parameters

#### 2.1.1 DALY calculation

The vaccination‐associated disease burden of each AEFI of interest was estimated using the composite DALY measure. The DALY sums the years lived with disability (YLD) for a health state (ie, living with a condition, disease, disability, or injury) with the years of life lost (YLL) due to premature mortality; thus, time is the metric for both morbidity and mortality. One DALY is equivalent to one lost year of life in perfect health.^3^, ^21^YLL and YLD are computed from a number of essential parameters^3^, ^22^:

*DALY =*: *YLL + YLD*
*YLL =*: *No. deaths × life expectancy at age of death*
*YLD =*: *No. events × disability weight × duration*

#### 2.1.2 Disability weights and durations

Disability weights encode the severity of the health outcome and can be obtained from professional or lay populations using a variety of preference elicitation methods;^23^ the current Global Burden of Disease (GBD)^10^ approach is to use general public survey respondents.^17^ The disability weight is on a scale from 0 (perfect health) to 1 (death). If weights are unavailable from existing databases or the relevant literature, then proxy health outcomes for which weights exist need to be assigned, preferably through consultation with experts with appropriate medical knowledge or through elicitation from survey respondents.^24^ Disability durations are typically determined from literature review, or from clinical expert knowledge if suitable studies cannot be located (see Appendix Methods 4).

#### 2.1.3 Outcome trees/subsequent sequelae

In cases where the occurrence of a certain adverse event can precipitate recurrence of the same event or can increase the risk of severe sequelae later in life, an outcome tree (also known as disease progression pathway) can be specified to incorporate the risk, severity, and duration of subsequent health outcomes.^25^

#### 2.1.4 Mortality

Distinguishing mortality as a causal reaction to vaccination from coincidental death is crucial, given the extreme rarity of vaccination‐attributable death.^26^ For a comprehensive burden estimate, one should also compute YLL for any adverse event for which there is evidence for a non‐zero case‐fatality rate. The BoD framework can easily include estimation of YLL for deaths confirmed as an immediate adverse outcome, and of YLL due to premature mortality associated with development of a long‐term sequela following the AEFI (via definition of an outcome tree, with a specified case‐fatality ratio; eg, Mangen et al^25^). For YLL, life expectancies from standard life tables are additionally required.^3^

#### 2.1.5 Over/under‐reporting and under‐ascertainment

Determination of either background adverse event incidence or direct attribution of the number of events to vaccination is susceptible to over‐reporting (misclassification of cases on an electronic health record [EHR] database, or comparable system), under‐reporting (misclassification, or failure to report cases seeking health care), and to under‐ascertainment (“missing” cases; those do not seek health care).^27^ In the presence of the latter two, burden will be under‐estimated. These factors can be problematic for comparison of burden between adverse events, if the extent of under‐reporting/ascertainment differs between event types.

### 2.2 Selection of parameters for the example burden calculation

The single most important outcome required for computing the health burden of AEFI is *vaccination‐attributable event incidence*. By “vaccination‐attributable”, we do not make the strong assumption that the observed adverse event has a causal relationship with the vaccine itself, but merely that the event is associated with and occurs following administration of the vaccine. By “attributable”, we refer to the extent to which the event incidence is associated with vaccination, adjusting for the expected, or background incidence in the population.

The *vaccination‐attributable event incidence* can be measured by various means: for instance, via calculation of incidence rates prior to and post‐vaccination by querying an EHR database with linked date(s) of vaccination(s), through primary data collection via cohort or self‐controlled case series designs,^28^ or from published reports of event incidence. If only background incidence rates (irrespective of vaccination) are available, then vaccination‐attributable incidence can be inferred through application of appropriate relative risk estimates and risk window‐size to the background incidence (see Appendix Methods 2).

We detail below the choices made for the example burden computation.

#### 2.2.1 Setting

Our worked example computes the burden of selected adverse events associated with routine vaccinations administered to young children (<4 years of age) only. Based on the availability of background incidence rates for overlapping time periods (below), we chose the UK as the population setting and estimated burden for the arbitrarily chosen year 2005, among the <4‐year‐old population.

#### 2.2.2 Background event incidence rates

For ITP and febrile convulsions, published background incidence rates were located for recent periods (1990–2005 and 1999–2014) from 2 studies using the UK General Practice Research Database (Table 1). For the latter event, data on narrow age groups were reported. We computed vaccination‐attributable event incidence and YLD based on incidence rates within these narrow age groups, and then later aggregated to 2 wider age groups (2 to 12 months, and 13 months to <4 years) for reporting purposes. Note that age groups can be fine‐tuned to the target ages for vaccination within the routine vaccination schedule, if event incidence rate data are available at a suitable granularity, for instance by month of age.

#### 2.2.3 Relative risks of vaccination‐attributable event

We conducted a pragmatic literature search for relative risks or absolute risks (defined in terms of cases per vaccine dose) for each of the 6 relevant vaccine‐event pairs that were classified as known associations in the GRiP reference set.^19^ A single effect estimate (RR or absolute risk) for each vaccine‐event pair was then chosen for the worked example (Table 2).

**Table 2:** Parameters for years lived with disability (YLD) computations for the selected vaccine‐event pairs in the worked example

Vaccine‐Adverse Event Pair	Age Group	RR or Risk per 1 M Doses (95% CI)	Reference	DW	DD
DTaP‐ITP	12–19 mos 4–6 yrs	1.00 (0.21–4.81) 2.57 (0.53–12.37) [6 week window]	O'Leary et al, 2012^12^	0.159	5 weeks
MMR‐ITP	<18 yrs	12.5/1 M doses (11.8–13.2)	Cheng et al, 2015^13^	0.159	5 weeks
DTaP/wP‐Anaphylaxis	0+ yrs	5.14/1 M doses (1.06–15.01)	McNeil et al, 2016^14^	0.552^a^	1 day
MMR‐Anaphylaxis	<18 yrs	1.3/1 M doses (0.03–7.1)	Bohlke et al, 2003^15^	0.552^a^	1 day
MenC‐Anaphylaxis	0+ yrs	6.16/1 M doses (1.68–15.78)	McNeil et al, 2016^14^	0.552^a^	1 day
MMR‐Febrile convulsions	3 mo – <10 yrs	2.75 (2.55–2.97) [14‐day window]	Vestergaard et al, 2004^16^	0.263^b^	1 day

Abbreviations: DD, disability duration; DTaP, diphtheria/tetanus/acellular pertussis; DW, disability weight; ITP, idiopathic thrombocytopenic purpura; MenC, meningococcal C; MMR, measles/mumps/rubella.

a Proxy used: epilepsy: severe.^17^

b Proxy used: “epilepsy: less severe”;^17^ see main text and Appendix Methods 4.

The age groups for which published relative risks or risks per dose were available did not necessarily match the relevant ages within the UK vaccination schedule (see Table 2 and http://vaccine-schedule.ecdc.europa.eu/Pages/Scheduler.aspx). For instance, the selected study for the vaccine‐event pair DTaP‐ITP provided a relative risk estimate for the age group 4 to 6 years, but in the UK the DTaP booster is recommended to be given at 3 years 4 months of age. Therefore, we made the following assumptions regarding applicability of a given published RR (or risk) to a particular age group. First, the first 3 DTaP (infant) doses were all assumed to have the same relative risk of ITP; the RR based on 12 to 19‐month‐old children^12^ was used. Second, the published RR for DTaP‐ITP based on 4 to 6 year olds was applied to the age group receiving the fourth dose (3 years 4 months). Third, identical RRs/risks were used for both first and booster MMR doses, as separate estimates were not available from the selected studies.

#### 2.2.4 Disability weights and durations

We obtained disability weights from the most recent GBD study (GBD 2013),^17^ which updates and expands the set of weights elicited for GBD 2010.^29^ Proxy weights were selected (by the authors) for 2 of the 3 selected events. Disability durations were retrieved from published sources (Table 2); for anaphylaxis and febrile convulsions, duration was assumed to correspond to average stay in hospital.^30^, ^31^

#### 2.2.5 Complications/long‐term sequelae

For our selected events, the risk of developing sequelae is either small, or there is insufficient evidence for progression. For instance, for febrile convulsions, we excluded the risk of suffering recurrent seizures (small increased rate of recurrence; estimated at 19%^16^). For ITP patients with a low platelet count, complications (severe bleeding) occur only rarely.^32^ For simplicity, we excluded the potential burden from additional health outcomes from our YLD estimates.

In addition, for all of our selected vaccine‐event pairs, death is recognized as extremely rare, at least in industrialized countries (for instance, no deaths following vaccination‐associated anaphylaxis were observed in a large USA study^15^). For simplicity, we excluded mortality and thus YLL from the worked example.

Finally, sensitivity analyses to assess the impact of selected parameters on AEFI burden may be appropriate when there is uncertainty around the choice of an appropriate parameter value. An example is the choice between multiple proxies for a disability weight not present in available sources.

### 2.3 Computation of YLD

We estimated YLD for 2 age groups 2 to 12 months, and 13 months to <4 years. All estimates were for the year 2005. Point estimates and 95% uncertainty intervals (UIs) were computed using R statistical software.^33^ We tabulated YLD per vaccine‐event pair and age group, as well as YLD/1 000 000 (ie, adjusting for age‐group population size), and additionally computed estimates aggregated over vaccine type and age group. Computational details are provided in Appendix Methods 1, and example R code is provided in Appendix Methods 3.

## 3 RESULTS

Vaccination‐attributable incidence rates and YLD for the 3 selected adverse events (ITP, anaphylaxis, and febrile convulsions (Table 1) were computed stratified by age group and vaccine type (Table 3). These results are provided to indicate how AEFI burden estimates can be usefully reported; they should not be regarded as valid estimates, as data and parameters were selected to illustrate the computation only.

**Table 3:** Results of example years lived with disability (YLD) computations (both absolute YLD and YLD per million population), for the selected vaccine‐event pairs, UK 2005. Vaccination‐attributable event incidence rate is per 100 000 person‐years

Vaccine‐Adverse Event Pair	Age Group	Vaccination‐Attrib. Inc. Rate (95% UI)	YLD (95% UI)	YLD/1 000 000 (95% UI)
DTaP – ITP	2–12 mos	2.12 (0.59–4.78)	0.19 (0.05–0.43)	0.32 (0.091–0.73)
	13 m–<4 yrs	1.26 (0.32–3.16)	0.52 (0.13–1.31)	0.19 (0.049–0.48)
MMR‐ITP	13 m–<4 yrs	0.53 (0.51–0.55)	0.22 (0.21–0.23)	0.081 (0.078–0.084)
*ITP (all vaccines)*	*2 m to < 4 yrs*	*1.89 (1.01–3.48)*	*0.96 (0.51–1.76)*	*0.29 (0.15–0.53)*
DTaP/wP‐Anaphylaxis	2–12 mos	1.62 (0.65–3.27)	0.015 (0.006–0.029)	0.025 (0.010–0.049)
	13 m –<4 yrs	0.10 (0.01–0.33)	0.004 (0.001–0.013)	0.002 (0.000–0.005)
MMR‐Anaphylaxis	13 m–<4 yrs	0.15 (0.08–0.29)	0.006 (0.003–0.012)	0.002 (0.001–0.004)
MenC‐Anaphylaxis	2–12 mos	1.35 (1.19–1.53)	0.012 (0.011–0.014)	0.021 (0.018–0.023)
	13 m–<4 yrs	0.14 (0.12–0.17)	0.006 (0.005–0.007)	0.002 (0.002–0.003)
*Anaphylaxis (all)*	*2 m to < 4 yrs*	*0.88 (0.65–1.23)*	*0.044 (0.033–0.062)*	*0.013 (0.010–0.019)*
MMR‐Febrile convulsions	13 m–<4 yrs	58.3 (32.3–103)	1.14 (0.63–2.02)	0.42 (0.23–0.74)
	*2 m to < 4 yrs*	*47.8 (26.6–84.9)*	*1.14 (0.63–2.02)*	*0.42 (0.23–0.74)*

Table 3 shows estimated vaccination‐attributable event incidence rates for the UK in 2005, per vaccine‐event pair and age group (2–12 months and 13 months to <4 years). Based on the selected parameter values, vaccination‐attributable incidence rates ranged from 0.10/100 000 to 58.3/100 000 person‐years.

**Figure 1:**
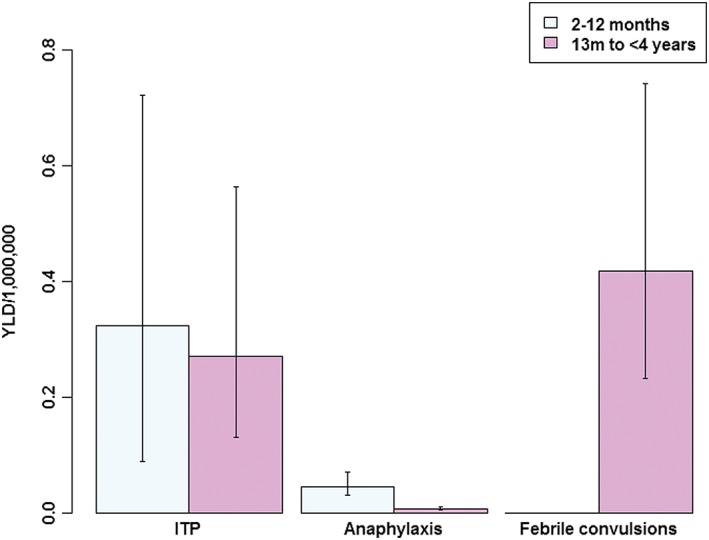
Estimated AEFI‐associated years lived with disability (YLD) per 1 000 000 persons, with 95% uncertainty intervals, by event and age group [Colour figure can be viewed at http://wileyonlinelibrary.com]

YLD, as well as YLD per 1 000 000 persons (to facilitate comparisons between age groups, across time. or between populations), are shown in Table 3 (see also Figure 1). The absolute morbidity burden ranged from 0.044 (95% UI: 0.033–0.062) to 1.14 (95% UI: 0.63–2.02).

## 4 DISCUSSION

We have presented methodology for estimating the morbidity burden associated with adverse events following vaccination, which we illustrated in the form of a worked example. Transparency of the computations involved the ability to compare the health burden of different AEFI using a common metric, and the expected ease of deployment beyond the 3 events we investigated are positive attributes of the applied method.

The extra insight provided by computing YLD (over vaccination‐attributable incidence only) for a specific vaccine‐event pair, is that YLD additionally takes into account the severity and duration (and possible longer‐term consequences) of the event. In our worked example, YLD/1 000 000 distinguished the population‐level health impact of vaccine‐event pairs with very similar attributable incidence rates (eg, DTaP‐ITP and DTaP‐Anaphylaxis for 2 to 12‐month‐old infants).

The worked example highlights the major limitation faced, namely data availability. Box 1 provides a summary of the data requirements for burden computation and options for specifying DALY parameters in the case that the preferred data source is unavailable.

**Box 1:** Data/parameter requirements, preferred sources, and possible alternatives for computation of the burden of adverse events following immunization using the DALY

Data/Parameter	Preferred Source	Alternative(s)
Vaccination‐attributable event incidence rate	Electronic health record database (EHR) linked to vaccination register	Systematic review of published relative risk estimates, applied to background incidence rates (from EHR or from systematic review of appropriate studies)
Outcome tree/subsequent sequelae, with associated risks	Published studies describing outcomes and quantified recurrence and/or progression risks	Clinical knowledge of appropriate medical experts
Disability weight	Disability weight database; weights elicited using standard methods	Proxy health outcome selected by medical experts and weights for proxies adopted or elicit new weights using established methods
Disability duration	Systematic review of studies reporting duration of health outcome	Clinical knowledge of appropriate medical experts

Literature search often did not yield relative risk estimates specific for the study population and/or age groups. It was difficult to find large studies (to provide sufficient statistical precision), or studies that were reasonably recent and/or geographically relevant. Conducting a meta‐analysis of published (relative) risks for each vaccine‐event pair, or estimation of the relative risks in the population might be preferred approaches. The granularity of the relative risks obtained from the literature was variable, with often very broad age groups defined, and for vaccines administered in multiple doses, separate estimates for each dose were not provided. Accordingly, we had to assume identical (relative) risks and risk periods for each dose. In addition, vaccine co‐administration—the norm for routine childhood immunization—complicate estimation of vaccination‐attributable incidence due to overlapping at‐risk periods. Clearly, application of published relative risks to background incidence rates, or use of absolute risks, requires numerous assumptions to be made about generalizability across time, setting, dose, and age group.

For one of our vaccine‐event pairs (DTaP‐ITP), relative risks were not significantly different from 1.0. Of course, statistical significance of the published RR depends on the presence of a real increased risk, as well as study power, as the number of outcomes is often very small. Obtaining relative risks—or better, vaccination‐attributable event incidence—directly from large EHR databases is a promising approach for improving precision. We stress that decisions regarding the acceptable power of candidate studies (from which parameters such as RR are obtained), and/or if meta‐analyses should be conducted, need to be made a priori.

Retrieving the required disability weights from published elicitation studies is a principled approach; however, 2 of our 3 selected events were not included in the most comprehensive and contemporary source available (GBD 2013)^17^, and consequently disability weights for proxy health outcomes needed to be chosen. Comparable collated data sources for disability durations do not exist, and although values can be located from diverse published sources (as we have done), a systematic review approach is clearly preferable, coupled with medical experts' review of the selected durations (also applicable to the selection of proxy disability weights). In general, missing data on either disability weights or disability durations (as both parameters have a linear relation with YLD) constitutes a major limitation to broad application of the methodology to a comprehensive set of AEFI. For the former parameter, selection of proxy health outcomes by medical experts is 1 option;^34^ a second option is to extend current databases through new elicitation of the missing disability weights.^24^

For illustration purposes, we focussed on selected events associated with routine (early) childhood vaccinations only. The AEFI burden associated with vaccinations received in adolescence and adulthood (eg, HPV, travel vaccinations, annual influenza jabs) is also of substantial interest, but the challenges in estimating burden are even greater, especially when estimating vaccination‐attributable event incidence from background incidence rates and relative risks. One‐time or ongoing medication use that may also cause the event of interest needs to be distinguished from vaccination (unlike for childhood vaccinations, there is normally no age schedule). Without information about a temporal relationship between either intervention and the event, correct attribution or adjustment is very difficult.

Vaccination of infants and children can influence the health state of the parent, for example as anxiety due to the occurrence of an adverse event and/or uncertainty of prognosis. We followed the BoD approach used for diseases and injuries and therefore ascribed burden only to the individual who received the vaccination.

For the current presentation of the BoD methodology, expert consultation was not needed. However, for real‐world application of the methods (see Box 1), we recommend that selection of all DALY parameters is guided by clinical knowledge of the adverse events of interest, and/or undergo review by medical experts (safety physicians), as the resulting burden estimates are crucially dependent on the appropriateness, acceptance, and validity of the parameter values chosen. A process of expert consultation is recommended at the data collection stage as well as for interpretation of the findings, for the dual purposes of ensuring scientific accuracy and credibility.^35^

DALYs are most usefully evaluated in context, for instance to create a ranking of diseases in terms of burden. For the burden of AEFI, estimates are meaningfully interpreted when they are compared with the burden of the disease(s) the vaccination prevents. If the population‐level disease burden averted by a vaccination programme is similarly quantified using the DALY—which importantly allows benefits and risks to be expressed in a common currency—then the current methodology may find useful application within a benefit‐risk monitoring platform. For vaccines which confer long‐term protection, the unit of time in which disease incidence is measured may need modification to provide a more appropriate benefit‐risk assessment, moving for instance from annual incidence to cumulative incidence. Finally, countries that currently use the QALY as basis for decision‐making regarding prevention initiatives could consider either to adapt the methodology to the QALY, or to switch to the DALY measure.

In conclusion, BoD methodology can feasibly be applied to estimate the health burden of adverse events following immunization, but interpretation of the findings must consider the quality, appropriateness, and accuracy of all data sources contributing to the DALY computation. Obtaining the required data is the single major barrier towards computing the burden of AEFI.

## ETHICS STATEMENT

The authors state that no ethical approval was needed.

## CONFLICT OF INTEREST

N.P. is an employee of the GSK group of companies and holds shares in the GSK group of companies as part of his employee remuneration. V.B. is an employee of the GSK group of companies and holds shares in the GSK group of companies as part of his employee remuneration. The remaining authors declare that they have no conflicts of interest.

## Supporting information

Data S1. Supporting information
Table A1. Selected disability weights and disability durations for the 3 example adverse events
