# Supplementary material for: Methodology for computing the burden of disease of adverse events following immunization
Source: Pharmacoepidemiol Drug Saf. 2018 Mar 24;27(7):724–30. doi: 10.1002/pds.4419 (PMC6055877; doi:10.1002/pds.4419)
Supplement: Supplementary file 1 — Data S1. Supporting information Table A1. Selected disability weights and disability durations for the 3 example adverse events [file PDS-27-724-s001.docx]

**SUPPLEMENTARY MATERIALS**

*To accompany the paper:*

**Methodology for computing the burden of disease of adverse events following immunization**

*Authors*: SA McDonald^1^, D Nijsten^1^, K Bollaerts^2^, J Bauwens^3,4^, N Praet^5^, M van der Sande^1^, V Bauchau^5^, T de Smedt^2^, M Sturkenboom^2,6^, S Hahné^1^

^1^ National Institute for Public Health and the Environment (RIVM), Bilthoven, Netherlands
^2^ P95 Pharmacovigilance and Epidemiology Services, Leuven, Belgium
^3^ University of Basel Children’s Hospital, Basel, Switzerland
^4^ Brighton Collaboration Foundation, Basel, Switzerland ^5^ GlaxoSmithKline Vaccines, Rixenart, Belgium
^6^ VACCINE.GRID Foundation, Basel, Switzerland

**APPENDIX METHODS 1
*Computational* *details***

Composite disease burden measures can be computed using specialised software (e.g., the DALY package for R; Devleesschauwer et al., 2013), custom software (Appendix Methods 3), or via a spreadsheet.

We estimated YLD for the two relevant age-groups: (i) the age-range encompassing the first set of vaccinations within the UK childhood scheme (2 to 12 months), and (ii) the age-group covering receipt of the third DTaP dose, the second meningococcal C vaccine (MenC) dose, and the first MMR dose (at between 12 and 13 months old), as well the DTaP and MMR boosters for three-year-olds (13 mos to <4 years). All estimates were for the year 2005, but given that background incidence rates were available aggregated over a period of 13 to 16 years (Table 1), YLD estimates for other years within the same period will be very similar, with variability due only to variation in population size and vaccination coverage.

The first step was to calculate the vaccination-attributable incidence rate for each event from either the background incidence rate (for ITP, febrile convulsions) or the risk per dose (anaphylaxis). See Appendix Methods 2 for a detailed description of the computation. As the UK schedule specifies three doses of DTaP (but only one of MMR and Men C) during infancy, three at-risk periods were defined for the DTaP–ITP pair according to the relevant window period sizes (6 weeks each; see Table 2) within the first 24 months of life (because the background incidence rate for ITP was available for <2 years). Dose-specific risks can be used if the relevant data are available; we assumed the RRs to be identical for all three DTaP infancy doses. The at-risk periods for the DTaP and MMR boosters were similarly defined according to the relevant window periods, for the background incidence age-groups 2–5 years (for ITP) and 13-24 mos (for febrile convulsions).

For anaphylaxis, no published background incidence rates were located; therefore, vaccination-attributable incidence was estimated based on the risk per million doses. We equated the number of doses to the number of vaccinated children in 2005, in turn estimated from the size of the UK birth cohort in 2005 (for doses 1-3 of DTaP, doses 1-2 of MenC), 2004 (dose 1 of MMR, dose 3 of MenC), or 2002 (booster doses of DTaP and MMR), multiplied by the relevant vaccination coverage value (see Appendix Methods 2, Eq. 8).

The second step was to compute YLD for each vaccine-event pair, stratified by age-group, based on the vaccination-attributable incidence adjusted for vaccination coverage (see Appendix Methods 2, Eq. 7) and the disability weight and duration (Table 2, main paper). Point estimates and 95% uncertainty intervals (UIs) were computed using R statistical software (R Development Core Team, 2015). R code is provided in Appendix Methods 3. Finally, we tabulated YLD vaccine-event pair and age-group, and additionally computed estimates aggregated over vaccine type and age-group.

**APPENDIX METHODS 2
*Calculation of vaccination-attributable adverse event incidence from published
relative or absolute risks***

First, consider that a relevant study of the risk of an adverse event following immunization reports a relative risk (RR), which is the ratio of the event incidence in a ‘window period’ following vaccination and the event incidence in a ‘control period’. To apply a RR to a given background event incidence rate, we need to estimate the incidence rates for the control period and in the window period.

In a 100% vaccinated population, the observed background event incidence rate, *inc_backgr_*, is (*n_vacc_ + n_control_*)/ total person-time. *n_vacc_* and *n_control_* refer to number of events in the risk ‘window’ post-vaccination of size *t* (in days) and the number of events in the ‘control period’, respectively. Both are unknown.

So, *inc_backgr_* is actually a weighted average; if the unit is one vaccinated person-year:

 *inc_backgr_ = inc_vacc_ * (t/365.25) + inc_control_ * ((365.25-t)/365.25)* (1)

Note that multiple doses within the period over which *inc_backgr_* is defined, can be easily handled by setting *t* to the size of the summed risk windows; for instance for *n* vaccine doses with associated risk window sizes *t_d_*:

 $t=\sum_{d}^{n} t_{d}$

If we let *w* = *(t/365.25)*, and rearrange to define *inc_vacc_* in terms of constants and one unknown:

 *inc_vacc_  =* $\frac{{inc}_{backgr}- ({inc}_{control} * (1-w))}{w}$ (2)

Given that RR = *(inc_vacc_ / inc_control_), we can re-arrange as:
 inc_control_ = (inc_vacc_ / RR)* (3)

and substitute in (2) to define *inc_vacc_* in terms of known values only:

 *inc_vacc_  =* $\frac{{inc}_{backgr}- ({inc}_{vacc}/RR * (1-w))}{w}$ (4)

after re-arrangement:

 *inc_vacc_  =* $\frac{{inc}_{backgr}}{(\frac{1-w}{RR}+w)}$ (5)

which is the incidence rate per vaccinated person-year, attributable to immunization.

Then, the expected number of adverse events associated with immunization in a populuation of *pop* vaccinated persons within one year of follow-up, where the period at risk is *t* days (and recall *w* = (*t*/365.25)) is:

 *n_vacc_* = *inc_vacc_ * w * pop* (6)

As the incidence rate *inc_vacc_* refers to a *vaccinated* population (more precisely, events per vaccinated person-year), it should be further adjusted for vaccination coverage *vc* (ie. if *vc* was 0%, then no adverse events could be attributed to immunization!).
 *n_vacc_* = *inc_vacc_ * vc * w * pop* (7)

This result is sufficient for input to the DALY calculation; we don’t need to do anything more.

Uncertainty in the RR can be incorporated by computing within a simulation (e.g., @Risk for Excel) or in a sampling/MCMC framework. This method can easily be generalised to use multiple RRs, for instance when reported separately for distinct risk windows (e.g., same day as immunization, 1-3 days following, 4-7 days following) and for multiple risk windows within a year (i.e., four DTP immunizations given within the first year of life).

Second, consider that the relevant study reports an absolute risk instead. For certain AEFI (eg. anaphylaxis), a RR is typically not reported because the event occurs immediately after immunization and can be considered to be caused by vaccination. Absolute risks are often reported as events per 1 million doses. In such a case, we can easily convert a risk provided as cases per million doses directly to the number of vaccination-attributable events, *n_vacc,_* as:

 *n_vacc_ = cases per 1M/1000000 * vc * pop* (8)

For instance, if in a given country the first dose of MMR is given at 12-13 months, the relevant population size (*pop*) can be approximated as size of birth cohort in the year previous (thus, for the DALY calculation only a single year-wide age-group needs to be specified). Note that this method does *not* use background incidence rate data.

**APPENDIX METHODS 3
*Example R code for AEFI burden computation***

## DALY calculation for AEFI

# call the function

source('/Users/anonymous/AEFI_DALY.R')

# create some data

n1 <- 6

n2 <- 10

BACKINC <- matrix(c(6.8),nrow = n1, ncol = n2)

BACKINC_LL <- matrix(c(4.9),nrow = n1, ncol = n2)

BACKINC_UL <- matrix(c(9.2),nrow = n1, ncol = n2)

backinc_py <- 100000 # denomintor of background incidence

dw <- 0.054 # disability weight

dur <- rep(28/365, n2) # duration

POP <- matrix(c(176839,172646,168033,165930,167756,173021),nrow=n1,ncol=n2) # pop size

COV <- matrix(c(.88,.85,.85,.82,.81,.82), nrow = n1, ncol = n2) # coverage

rr <- 5.48 # relative risk

rr_ll <- 1.61

rr_ul <- 18.64

rw <- 42 # lenght of risk window in days

daly_py <- 100000 # standardisation of population size

niter <- 10000 # number of MC iterations

#....and test the function

AEFI_DALY(BACKINC, BACKINC_LL, BACKINC_UL, backinc_py, POP, COV, rr, rr_ll,

rr_ul, rw, dw, dur, daly_py, niter)

## DALY calculation for AEFI

# filename “AEFI_DALY.R”

AEFI_DALY <- function(BACKINC, BACKINC_LL, BACKINC_UL, backinc_py, POP, COV,

rr, rr_ll, rr_ul, rw, dw, dur, daly_py, niter)

{

# Function AEFI_DALY: computation of DALYs for AEFI

# INPUTS:

# BACKINC = matrix of size m x n of background inc. rates, m=years, n=age groups

# BACKINC_LL = matrix of size m x n of the lower limits of the 95% CI of the
# background inc. rates, m = years, n = age groups

# BACKINC_UL = matrix of size m x n of the upper limits of the 95% CI of the
# background incidence rates, m = years, n = age groups

# backinc_py = denominator of the background incidence (xx person years)

# POP = matrix of size m x n of population size, m = years, n = age groups

# COV = matrix of size m x n of vaccination coverage, m = years, n = age groups

# rr = scalar containing the relative risk (RR) of the AEFI

# rr_ll = scalar containing the lower limit of the 95% CI of the relative risk

# rr_ul = scalar containing the uppler limit of the 95% CI of the relative risk

# rw = duration of the risk window in days

# dw = scalar containing disability weight

# dur = vector of length n containing avg dur. of disease/event by age-group (days)

# daly_py = standardize population size, e.g. DALY per 100.000 person-years
# (daly_py = 100.000)

# niter = number of iterations

#

# OUTPUTs:

#

# 1. INC ATTR TO VACCINE

# .....

# 2. DALY (tot population)

# ....

# 3. DALY (standardized population size)

# YLDz_by_yr_age_median = DALY (YLD) by year x age group, median

# YLDz_by_yr_age_p025 = DALY (YLD) by year x age group, percentile 0.025

# YLDz_by_yr_age_p975 = DALY (YLD) by year x age group, percentile 0.975

#

# YLDz_by_age_median = DALY (YLD) by age group, median

# YLDz_by_age_p025 = DALY (YLD) by age group, percentile 0.025

# YLDz_by_age_p975 = DALY (YLD) by age group, percentile 0.975

#

# YLDz_by_yr_median = DALY (YLD) by calendar year, median

# YLDz_by_yr_p025 = DALY (YLD) by calendar year, percentile 0.025

# YLDz_by_yr_p975 = DALY (YLD) by calendar year, percentile 0.975

#

# YLDz_overall_median = DALY (YLD) overall, median

# YLDz_overall_p025 = DALY (YLD) overall, percentile 0.025

# YLDz_overall_p975 = DALY (YLD) overall, percentile 0.975

#

#

# support functions: betaparams()

#

# latest update: 07/12/2016

# method of moments: from mean and 95% CI to parameters beta distribution -beta(a, b)

betaparams <- function(m,ll,ul) {

range <- ul - ll

SD <- (range /(2*1.96))

a <- (m^2-m^3-m*SD^2)/SD^2; b <- (m-2*m^2+m^3-SD^2+m*SD^2)/SD^2

return(c(a = a,b = b))

}

# calculate YLD for AEFI

n1 <- dim(BACKINC)[1]

n2 <- dim(BACKINC)[2]

# preallocate 3-D output arrays (n1=years; n2=age groups; n3=niter)

incvacc = array(NA, dim=c(n1,n2,niter))

YLD = array(NA, dim=c(n1,n2,niter))

YLD_z = array(NA, dim=c(n1,n2,niter))

for(i in 1 : n1) {

for(j in 1 : n2) {

## 1. Monte Carlo sampling: sample from distributional forms assumed for
 background incidence rate and relative risk

# 1.a Background incidence: assume Beta distribution:

t <- betaparams(BACKINC[i, j] / backinc_py, BACKINC_UL[i, j] / backinc_py,

BACKINC_LL[i, j] / backinc_py)

a_ij <- t[1]; b_ij <- t[2]

backinc_sampled <- rbeta(niter,a_ij,b_ij) * backinc_py

# 1.b Relative risk: assume normal distribution for log-relative risk (ie.
 # log-normal); need to approximate variance using supplied 95% CI

approx_sd <- (log(rr_ul) - log(rr_ll))/(2 * 1.96)

rr_sampled <- rlnorm(n=niter,meanlog=log(rr),sdlog=approx_sd)

## 2. derive vaccination-associated incidence rate

rw_yr <- (rw/365.25)

incvacc_sampled <- backinc_sampled/ (((1-rw_yr)/rr_sampled) + rw_yr)

incvacc[i,j, ] <- incvacc_sampled

## 3. calculate years lived with disability in the total population

YLD_sampled <- (incvacc_sampled/backinc_py*COV[i,j]*POP[i,j]*rw_yr)*dw*dur[j]

YLD[i,j, ] <- YLD_sampled

## 4. standardize population size

YLD_z_sampled <- (YLD_sampled / POP[i, j]) * daly_py

YLD_z[i,j, ] <- YLD_z_sampled

}

}

# SLICE AND DICE to OUTPUT (outcomes: vaccine attributable incidence, DALY, DALY per standardized population), (estimates: median, p0.25, p0.975), by (time x age, time, age, overall)

# 1.vacc attributable incidence

# incvacc by age x year

incvacc_by_yr_age_median <- round(apply(incvacc, 1:2, quantile, .5, names = FALSE),3)

incvacc_by_yr_age_p025 <- round(apply(incvacc, 1:2, quantile, .025, names = FALSE),3)

incvacc_by_yr_age_p975 <- round(apply(incvacc, 1:2, quantile, .975, names = FALSE),3)

# incvacc by age

incvacc_agg <- apply(incvacc, 2:3, sum, names=FALSE)

incvacc_by_age_median <- round(apply(incvacc_agg, 1, quantile, .5, names = FALSE),3)

incvacc_by_age_p025 <- round(apply(incvacc_agg, 1, quantile, .025, names = FALSE),3)

incvacc_by_age_p975 <- round(apply(incvacc_agg, 1, quantile, .975, names = FALSE),3)

# incvacc by year

incvacc_agg <- apply(incvacc, c(1,3), sum, names=FALSE)

incvacc_by_yr_median <- round(apply(incvacc_agg, 1, quantile, .5, names = FALSE),3)

incvacc_by_yr_p025 <- round(apply(incvacc_agg, 1, quantile, .025, names = FALSE),3)

incvacc_by_yr_p975 <- round(apply(incvacc_agg, 1, quantile, .975, names = FALSE),3)

# incvacc overall

incvacc_agg <- apply(incvacc, 2:3, sum, names=FALSE)

incvacc_agg <- apply(incvacc_agg, 2, sum, names=FALSE)

incvacc_overall_median <- round(quantile(incvacc_agg,prob=c(0.5)),3)

incvacc_overall_p025 <- round(quantile(incvacc_agg,prob=c(0.025)),3)

incvacc_overall_p975 <- round(quantile(incvacc_agg,prob=c(0.975)),3)

# 2.YLD total population

# YLD by age x year

YLD_by_yr_age_median <- round(apply(YLD, 1:2, quantile, .5, names = FALSE),3)

YLD_by_yr_age_p025 <- round(apply(YLD, 1:2, quantile, .025, names = FALSE),3)

YLD_by_yr_age_p975 <- round(apply(YLD, 1:2, quantile, .975, names = FALSE),3)

# YLD by age

YLD_agg <- apply(YLD, 2:3, sum, names=FALSE)

YLD_by_age_median <- round(apply(YLD_agg, 1, quantile, .5, names = FALSE),3)

YLD_by_age_p025 <- round(apply(YLD_agg, 1, quantile, .025, names = FALSE),3)

YLD_by_age_p975 <- round(apply(YLD_agg, 1, quantile, .975, names = FALSE),3)

# YLD by year

YLD_agg <- apply(YLD, c(1,3), sum, names=FALSE)

YLD_by_yr_median <- round(apply(YLD_agg, 1, quantile, .5, names = FALSE),3)

YLD_by_yr_p025 <- round(apply(YLD_agg, 1, quantile, .025, names = FALSE),3)

YLD_by_yr_p975 <- round(apply(YLD_agg, 1, quantile, .975, names = FALSE),3)

# YLD overall

YLD_agg <- apply(YLD, 2:3, sum, names=FALSE)

YLD_agg <- apply(YLD_agg, 2, sum, names=FALSE)

YLD_overall_median <- round(quantile(YLD_agg,prob=c(0.5)),3)

YLD_overall_p025 <- round(quantile(YLD_agg,prob=c(0.025)),3)

YLD_overall_p975 <- round(quantile(YLD_agg,prob=c(0.975)),3)

# 3. YLD standardized

# YLD by age x year

YLDz_by_yr_age_median <- round(apply(YLD_z, 1:2, quantile, .5, names = FALSE),3)

YLDz_by_yr_age_p025 <- round(apply(YLD_z, 1:2, quantile, .025, names = FALSE),3)

YLDz_by_yr_age_p975 <- round(apply(YLD_z, 1:2, quantile, .975, names = FALSE),3)

# YLD by age

YLD_z_agg <- apply(YLD_z, 2:3, sum, names=FALSE)

YLDz_by_age_median <- round(apply(YLD_z_agg, 1, quantile, .5, names = FALSE),3)

YLDz_by_age_p025 <- round(apply(YLD_z_agg, 1, quantile, .025, names = FALSE),3)

YLDz_by_age_p975 <- round(apply(YLD_z_agg, 1, quantile, .975, names = FALSE),3)

# YLD by year

YLD_z_agg <- apply(YLD_z, c(1,3), sum, names=FALSE)

YLDz_by_yr_median <- round(apply(YLD_z_agg, 1, quantile, .5, names = FALSE),3)

YLDz_by_yr_p025 <- round(apply(YLD_z_agg, 1, quantile, .025, names = FALSE),3)

YLDz_by_yr_p975 <- round(apply(YLD_z_agg, 1, quantile, .975, names = FALSE),3)

# YLD overall

YLD_z_agg <- apply(YLD_z, 2:3, sum, names=FALSE)

YLD_z_agg <- apply(YLD_z_agg, 2, sum, names=FALSE)

YLDz_overall_median <- round(quantile(YLD_z_agg,prob=c(0.5)),3)

YLDz_overall_p025 <- round(quantile(YLD_z_agg,prob=c(0.025)),3)

YLDz_overall_p975 <- round(quantile(YLD_z_agg,prob=c(0.975)),3)

llist <- list(incvacc_by_yr_age_median = incvacc_by_yr_age_median, incvacc_by_yr_age_p025 = incvacc_by_yr_age_p025, incvacc_by_yr_age_p975 = incvacc_by_yr_age_p975,

incvacc_by_age_median = incvacc_by_age_median, incvacc_by_age_p025 = incvacc_by_age_p025, incvacc_by_age_p975 = incvacc_by_age_p975,

incvacc_by_yr_median = incvacc_by_yr_median, incvacc_by_yr_p025 = incvacc_by_yr_p025, incvacc_by_yr_p975 = incvacc_by_yr_p975,

incvacc_overall_median = incvacc_overall_median, incvacc_overall_p025 = incvacc_overall_p025, incvacc_overall_p975 = incvacc_overall_p975,

YLD_by_yr_age_median = YLD_by_yr_age_median, YLD_by_yr_age_p025 = YLD_by_yr_age_p025, YLD_by_yr_age_p975 = YLD_by_yr_age_p975,

YLD_by_age_median = YLD_by_age_median, YLD_by_age_p025 = YLD_by_age_p025, YLD_by_age_p975 = YLD_by_age_p975,

YLD_by_yr_median = YLD_by_yr_median, YLD_by_yr_p025 = YLD_by_yr_p025, YLD_by_yr_p975 = YLD_by_yr_p975,

YLD_overall_median = YLD_overall_median, YLD_overall_p025 = YLD_overall_p025, YLD_overall_p975 = YLD_overall_p975,

YLDz_by_yr_age_median = YLDz_by_yr_age_median, YLDz_by_yr_age_p025 = YLDz_by_yr_age_p025, YLDz_by_yr_age_p975 = YLDz_by_yr_age_p975,

YLDz_by_age_median = YLDz_by_age_median, YLDz_by_age_p025 = YLDz_by_age_p025, YLDz_by_age_p975 = YLDz_by_age_p975,

YLDz_by_yr_median = YLDz_by_yr_median, YLDz_by_yr_p025 = YLDz_by_yr_p025, YLDz_by_yr_p975 = YLDz_by_yr_p975,

YLDz_overall_median = YLDz_overall_median, YLDz_overall_p025 = YLDz_overall_p025, YLDz_overall_p975 = YLDz_overall_p975)

return(llist)

}

**APPENDIX METHODS 4**

**Table A1.** Selected disability weights and disability durations for the three example adverse events

| **Adverse event** | **Disability weight** | **Disability duration** |
| --- | --- | --- |
| Idiopathic thrombocytopenia purpura (ITP) | Thrombocytopenia purpura: 0.159  [source: Salomon et al., 2015] | 2-8 weeks (max. 6 months) [sources: <http://patient.info/health/immune-thrombocytopenia-leaflet>; <http://www.netdoctor.co.uk/conditions/heart-and-blood/a1176/idiopathic-thrombocytopenic-purpura-itp/>] |
| Anaphylaxis | *Proxy:*  Epilepsy: severe: 0.552  [source: Salomon et al., 2015] | 1 day  [source: Ginsberg et al., 2015] |
| Febrile convulsions/seizures | *Proxies:*  Epilepsy: less severe: 0.263  [source: Salomon et al., 2015] | 1 day [source: Oluwabusi & Sood, 2012] |

**References**

Ginsberg GM, Eidelman AI, Shinwell E, Anis E, Peyser R, Lotan Y. Should Israel screen all mothers-to-be to prevent early-onset of neonatal group B streptococcal disease? A cost-utility analysis. Israel J Health Policy Res. 2013; 2(1): 6.

Oluwabusi T, Sood SK. Update on the management of simple febrile seizures: emphasis on minimal intervention. Curr Opin Pediatr. 2012; 24(2): 259-65.

Salomon JA, Haagsma JA, Davis A, et al. Disability weights for the Global Burden of Disease 2013 study. The Lancet Global Health 2015; 3(11): e712-23.
